# Sexual and reproductive health correlates of polysubstance use among female adolescents who sell sex in the southwest of China

**DOI:** 10.1186/s13011-020-00302-5

**Published:** 2020-08-17

**Authors:** Xu-Dong Zhang, Jun Zhang, Ren-Sheng Xie, Wei-Hong Zhang

**Affiliations:** 1 Yunnan Research Centre for Healthcare Management, School of Management and Economics, Kunming University of Science and Technology, Kunming, China; 2 China-UK Research Centre for Reproductive Health (Yunnan Province), The Affiliate Hospital, Kunming University of Science and Technology, Kunming, China; 3 Public Health Research Centre, Tsinghua University, Beijing, China; 4 School of Medical Humanities, Zunyi Medical University, No.6 Xue Fu Xi Lu Rd., Xinpu District, Zunyi, 563006 Guizhou Province China; 5 International Centre for Reproductive Health, Department of Public Health and Primary Care, Ghent University, Ghent, Belgium; 6 School of public Health, Université Libre de Bruxelles (ULB), Brussels, Belgium; 7 Research Laboratory for Human Reproduction, Faculty of Medicine, ULB, Brussels, Belgium

**Keywords:** Adolescents, Female sex worker, Substance use, Sexual and reproductive health, China

## Abstract

**Background:**

Substance use and adverse sexual and reproductive health (SRH) outcomes continue to be significant threats to female adolescents’ health and wellbeing, particularly to these marginalized subpopulations. Our research aimed to tackle knowledge gaps regarding the prevalence of concurrent substance use including alcohol, tobacco, illicit drug among Chinese adolescent female sex workers (AFSWs), while to assess the correlates between substance use and SRH risks; the needs for comprehensive SRH services were also examined in this study.

**Methods:**

A cross-sectional study enrolled 310 AFSWs aged 15–19 years by using cluster sampling method in Kunming, China. Descriptive analysis was employed to characterize the participants who were regular-alcohol users, regular-tobacco users, illicit drug users and polysubstance users. Multivariable logistic regression analysis was performed to detect the SRH correlates of regular-alcohol use, regular-tobacco use, illicit drugs and polysubstance use respectively.

**Results:**

There is a high prevalence of regular-alcohol drinking (83%, 257/310) among AFSWs, with 44% (136/310) smoking cigarettes regularly and 9% using illicit drug (27/310) in the past year. In multivariate analysis, AFSWs who had middle and high school education, had higher monthly income, experienced of sexual and gender based violence (SGBV) and prior abortion, and regular-tobacco smoking were associated with increased odds of regular-alcohol drinking; engaging in unprotected sex while drunk, having STIs symptoms and using illicit drugs were significantly associated with regular-tobacco smoking; while AFSWs who had an illicit drug using- intimate partner, experienced forced sexual initiation, accessed unsafe medical providers for STIs treatment were associate with increased odds of illicit drug use. Moreover, 35% (105/298) AFSWs sought unsafe medical care for STIs treatment, or no treatment at all, among them, majority were using polysubstance (87%; 91/105).

**Conclusions:**

Our findings reveal combined threats of substance use to AFSWs’ SRH and wellbeing in China, this study emphasises that the coordinated efforts are needed to integrate SRH promotion and harm reduction service across sectors, and not only fragmented measures. An effective response should include an agreed framework, indicators and targets supported by political will, solid leadership and policy reform to deal with AFSWs’ overlapping vulnerabilities in a systematic way.

## Background

Adolescence is a critical stage of life characterised by rapid biological, emotional and social development. There are about 1.2 billion adolescents aged 10–19 years in the world today; 13% (154,222,000) of adolescent women aged 15–19 years live in China [1]. Now themes regarding adolescents’ sexual and reproductive health (SRH) and rights, substance use prevention and treatment are higher on the global development agenda than ever before, and are currently featured on the the 2030 Agenda of Sustainable Development Goals (Target 3.a, Target 3.5, Target 3.7, Target 4.5 and Target 5.6) because of the far-reaching implications for their health and for social and economic development [2]. A critical new priority at the heart of the updated *Global Strategy for Women’s Children’s and Adolescents’ Health* (2016–2030) is the focus on adolescents. This Global Strategy also brings a broader concept of adolescent SRH by including HIV and other sexually transmitted infectious (STIs), non-communicable diseases (NCDs), and mental and substance use disorders [3].

Substance use among adolescents can lead to increased risk of STIs including HIV, vehicular fatalities, juvenile delinquency, and other NCDs problems associated with physical and mental health [4]. Global Burden of Disease Study brought to light, in 2017, smoking and alcohol use disorders were the third and tenth leading contributor to the number of deaths and percentage of age-standardized disability-adjusted life-years (DALYs) in China [5]. Across the 10–24 year age group, alcohol misuse was the risk factor contributing to the highest proportion (7%) of DALYs, and alcohol misuse and drug use disorders were the risk factors for DALYs for Chinese young people aged 20–24 years; furthermore, unsafe sex was the most significant risk factor for DALYs lost for adolescents aged 15–19 years [6]. Meanwhile, adverse SRH consequences disproportionately affect female adolescents who use alcohol and tobacco [6], for example, maternal smoking during pregnancy is a particular risk factor for poor fetal growth as well as later-life illness in offspring [4]; alcohol use during puberty adversely affects the maturation of the reproductive system while greater use of alcohol in pregnancy has prominent intergenerational harms in the form of fetal alcohol syndrome [4].

Although most in need, a scarcity of data limits of substance use and associated SRH risks concerning marginalised or vulnerable adolescents are a major challenge [7]. Across developing countries, there are underreporting gaps in data and research related to the adolescents’ substance use behaviours and SRH outcomes, that situation is more specifically in China [8]. Moreover, the Guttmacher Institute report on adolescent women’s need for and use SRH health services in 70 developing countries showed that only nine Asian countries but China had data available [8]. In China, all forms of sex work are illegal and highly stigmatised; sex workers are often treated as quasi-criminals and can receive administrative penalties of 15-day detention or fines [9]. Adolescents who both sell sex and use drugs face heavier penalties. Aside from political tension and criminalisation, the social norms and cultural taboos usually bring more ethical challenges into SRH promotion actions and research in marginalized adolescents [10], consequently adolescent female who sell sex or/and use drugs as a class remains hidden and ignored in China [11, 12]. The lack of basic knowledge hinders effective responses for the adolescent female sex workers (AFSWs)’ SRH and wellbeing. To make these marginalized adolescents visible in policy design and programme initiatives, specific investigation is needed to record the patterns substance use including alcohol, illicit drugs and tobacco, to explore the relations between substance use and the broad SRH risks including HIV/STIs, unmet need for modern contraception, abortion, sexual and gender-based violence (SGBV), SRH care seeking behaviours and specific service needs.

In 1977, the American pathologist and psychiatrist George Engel first conceptualized and introduced the biopsychosocial model that combines biological, psychological and social factors to understand health and illness [13]. This approach is rooted in ‘general systems theory’, which states that a system is characterised by the interactions of its components. As an applied multidisciplinary science, biopsychosocial model is well applied by specialist societies in obstetrics and gynaecology areas [14]; meanwhile a variety of studies used the biopsychosocial model for examining the roles of environment and personal values (i.e., psychosocial factors) in the onset of adolescent risk-taking behaviour, for example, the initiation of substance use [15]. Now the *Global Strategy for Women’s Children’s and Adolescents’ Health* calls for a multisector approach to improve the health and wellbeing of adolescents. Thus, the biopsychosocial model provides a valuable basis for our research to explore how social-economic, social-environmental, substance use and broader SRH factors interact in AFSWs’ underlying vulnerabilities, and ultimately, determine key areas for collaborative intervention action.

Our research aimed to tackle knowledge gaps regarding the prevalence of concurrent alcohol, tobacco, illicit drug and polysubstance use among Chinese AFSWs, as well as to assess the correlates between these substance use and SRH risks respectively; the needs for comprehensive SRH services were also examined in this study.

## Methods

### Study setting and population

Yunnan Province on China’s southwestern border is close to the ‘Golden Triangle’, a major international business and drug trafficking route. This province has a long and evolving history of drug trade as a notorious centre in China, seizures made by Yunnan along the ‘Golden Triangle’ route accounted for 83% of national quantities of heroin, ketamine and amphetamines seized in 2019 [16]. The health consequences documented include a relatively high HIV-1 prevalence and other STIs in China [17]; between 2010 and 2015, more than 10,000 new HIV/AIDS diagnoses occur among women aged 15–49 years [18]. Meanwhile Yunnan is also the largest grower of tobacco leaf and has cigarette industry in China, roughly 70% of Yunnan Province’s annual tax revenue is collected from the tobacco industry [19]. This study was conducted in Kunming city, which is the largest economic centre and the capital city of Yunnan Province, has an estimated population of 7.2 million. It is estimated that about 10,200 FSWs were active in Kunming (approximately 0.33% of Kunming’s female population) in 2013, with about 7900 (77%) concentrated in urban areas [20], the proportion estimates of AFSWs range from 15 to 25% [21]. Due to the notable paucity of studies and official data reporting HIV/STIs prevalence and population estimation of FSWs after 2015 in China [22], as well as data scarcity on the changing patterns of substance use among AFSWs, in June 2020, we conducted in-depth interviews among 5 key informants from two non-governmental organizations supporting FSWs and the Kunming Chinese Centre of Disease Control. Interviewees reported that the number of less-educated young and out-of-school adolescent women involved in sex trade was increased than 2013. In the past decade, annually millions of people from countryside move to big cities across China driven by the government’s urbanization plan. Overall, the number of rural-to-urban young women involved in providing sexual services is often greater than their urban residents counterparts. Potential motivation is link to economic hardship or the lure of fast and easy earnings, needs of sharing rent or building relationship, and limited employment opportunities for less-educated female migrants. However, sex work no longer remains a full-time job for many AFSWs, they tend to see the commercial sex as a temporary or occasional arrangement without perceiving themselves to be sex workers. While the types of worksites for soliciting clients are shifting due to the popularization of smartphone and social media platforms. Thus the observed number of AFSWs who are working at traditional entertainment establishments (e.g., karaoke, night club, dancing hall, disco, bar) or personal service sectors (e.g., hair washing room, hair salon, massage parlour, sauna, restaurant, hotel) has declined over time. On the other hand, based on interviewees’ routine work contact, the number of cases of HIV/AIDs or other STIs (such as syphilis) among these adolescent women involved in all forms of commercial sex has grown since 2018, suggesting the SRH risks were more continually to occur.

Our study was designed to enrol consenting females aged 15–19 years who self-reported to have received money or gifts in exchange for sex from one or more paying clients in the past 6 months, and were living in urban sites during the period of study. This study referred enrolled adolescent females engaged in commercial sex as AFSWs.

### Study design

This was a cross-sectional study using the Chinese version of semi-structured questionnaires which adopted questions regarding knowledge of family planning, knowledge of HIV/STIs, sexual relationship and behaviour from the ‘WHO questionnaire for young people’ [23] and the ‘Indonesia Demographic and Health Survey Questionnaire for Young Adult Reproductive Health’ [24]; some questions regarding SGBV and relationship control were adopted from a cohort study of FSWs in Kenya [25]. All three original instruments had been tested for reliability and validity. Two pilot tests using the draft questionnaire were conducted among key informants (leaders of FSWs’ support organizations/frontline clinic doctors/senior peer educators/outreach workers) and 14 AFSWs.

### Data collection

The study was implemented in collaboration with Kunming-based, FSW-led, grassroots organizations and the district level Chinese Centres of Disease Control. A total of 101 locations in four urban districts of Kunming were identified as places where young FSWs worked frequently. A stratified random sampling was planned, however the ongoing nationwide police crackdown on the sex industry made it impossible; instead, a one-stage cluster sampling method was employed to recruit study participants between September 2012 and February 2013. The initial stage of sampling involved 27 clusters (locations), which were randomly selected from the 101 identified locations, proportionate to the total number of locations in each district area. Potentially eligible women were recruited from the 27 clusters.

Six health workers (clinical doctors/nurses selected from district level health facilities) and six peer educators (FSWs from local grassroots organizations) were trained as interviewers about the ethical issues, study purpose, survey procedures and questionnaire administration. Using a semi-structured questionnaire, face-to-face interviews were conducted at 27 locations, and each interview took 40–50 min to complete.

### Study measures

For the purposes of this analysis, we focused on regular-alcohol drinking, regular-tobacco smoking, illicit drug and polysubstance use as four dependent variables; while based on the biopsychosocial model framework, independent variables were selected and grouped into the following five dimensions, namely: socio-demographic characteristics, sex work, sexual behaviours, sexual health, and concurrent substance use.

Since there is some scientific and legal ambiguity about the distinctions between substance ‘misuse’ and ‘abuse’, in this study, the neutral term ‘substance use’ was used. Substance types and use patterns were measured including alcohol, tobacco, illicit drugs, and polysubstance in the past 12-month period before the survey date.

Illicit drug use was defined as the self-reported use any sort of heroin, methamphetamine pill, crystalline methamphetamine, marijuana, ketamine and ecstasy.

Alcohol use was assessed by the self-reported frequencies of alcohol drinking, and was dichotomised as abstainer or occasional-alcohol drinking (once per week or less) versus regular-alcohol drinking (daily or more than twice a week).

Tobacco use was defined as self-reported frequencies of cigarette smoking, and dichotomised as abstain or occasional-tobacco smoking (less than 1–2 times monthly), and regular-tobacco smoking (daily or more than twice a week).

Participant, who was regularly using alcohol, while concurrently using any sort of illicit drug or regularly using tobacco, was characterized as polysubstance use.

The term of ‘SGBV’ referred to any sex worker self-reporting an act of violence from an intimate male partner (husband, boyfriend, or non-paying partner) or paying male clients, and which resulted in, or was likely to result in, physical, sexual harm or suffering to participants, whether occurring in the work place or in their private life.

The term of ‘unmet need for modern contraception’ was defined as not currently intending to get pregnant, but neither using any modern contraception (sterilisation, oral contraceptive pill, intra-uterine device, diaphragm, injection, emergency contraception, or implant), nor consistently using condom with any sexual partners.

The term of ‘inconsistent condom use’ was defined as not always using condoms in the past month, including when drunk or under the influence of drugs with intimate male partners and/or male clients.

The term of ‘any self-reported symptom of STIs’ was included at least one symptom of unusual vaginal discharge, vaginal itching, frequent burning pain on urination, genital ulcers/scores.

The term of ‘safe medical care’ was defined as formally supervised by governmental health department and operated by registered and well-trained medical staff; on the contrary, ‘unsafe medical care’ was defined as seeking treatment at unregistered small clinic or from self-employed, unqualified health workers, or using self-medication, or no any treatment at all.

### Data management and analysis

Interviewers were paired and logical errors in the questionnaires were cross-checked on a daily basis. Paper-based data was double-entered using EpiData version 3.1 by trained staff. Identified discrepancies were corrected in line with the original questionnaires.

Analyses were performed using Stata version 13.0 (Stata Corp, College Station, Texas, USA). The ability to correct for complex sample designs in analyzing survey data is an important advantage of Stata. Using clustering samples in this study might reduce the accuracy of estimates (increases the standard error) rather than pure random samples. Therefore, our primary analytic approach was to adjust the potential intra-class correlation within 27 clusters (seeds), the syntax of ‘svyset ‘command was used to describe the sample design, then the’ ‘svy:’ prefix was used before other commands of probit analysis. Survey-adjusted odds ratios are presented for the initial bivariate model and the final multivariate model, respectively.

Descriptive analysis was employed to characterize the participants. Pearson’s Chi-squared tests and Wilcoxon rank sum tests were used to assess associations between independent variables and dependent variables. Dependent variable with *p*-value less than 0.05 in descriptive analysis were included in the bivariate logistic analysis, and variables at the *p < 0.05* significant level in bivariate analysis were selected for inclusion in the multivariable model by using a stepwise forward-fitting approach; all independent variables had been identified the multi-collinearity (variance inflation factor < 10) before entering into multivariable logistic analysis. Independent variables that did markedly alter the model’s fit were removed from the model, and only the variables that had *p*-values less than 0.05 were retained in the final multivariable model. The Akaike information criterion (AIC) has been used as a measure of goodness-of-fit during the model-selection procedure, the multivariable model with the smallest value of AIC was considered as the final model. Missing responses on selected variables were not included in our analysis.

### Ethics

Ethics approvals and permissions to the study protocol, informed consent forms, information sheets and other requested documents, or any subsequent modifications—were obtained from the ethical committee of the Kunming Public Health Bureau. All interviewers received training in research ethics, including non-judgmental interview skills and confidentiality.

None of the participants were under the age of 15 years old in our study, all participants were clearly informed about the study objectives, the confidential nature of information collected, and their rights of voluntary participation including refusal to answer questions and withdrawal from the study. All participants provided their written informed consent before the interview. For participants who were under the age of 18, given the fact that they were estranged from the parents and living independently in daily life, under this circumstance, adolescents were more knowledgeable about their own situation than their parents, and their priorities and preferences may be at odds with their family and community of origin. In addition, the results of prior focus group discussions indicated that it was neither feasible nor appropriate to obtain parental/guardian consent because there was a risk of breaking their confidentiality, and posing potential barriers hindering their inclusion in this research, a formal written informed consent was additionally obtained from an adult peer selected by the respective participant. Confidentiality was maintained during the recruitment process, questionnaire administration and data storage.

Two existing drop-in centres operated by FSW-led grassroots organizations provided an informal safe space for victims of SGBV to join group or personal activities including medical treatment and peer support. The centres organised referral for clinical care services when indicated.

## Results

A total of 378 adolescent FSWs were approached. Of these, 342 women (90%) were eligible and consented to participate and 310 (91%) completed the interview and included in the analysis.

### Descriptive statistics of socio-demographic characteristics

The median self-reported age of the study participants was 19 years (inter-quartile range 18–19 years). 54% of the AFSWs had average of 9–12 years of secondary education and 18% had 13–15 years of secondary education. Over half the participants were married or cohabiting (53%; 165/310). Most AFSWs were new rural-to-urban migrants (83%; 257/310). The majority of the participants also reported that they newly entered sex work industry for less than 6 months (62%; 191/310) (Table 1).

**Table 1:** Socio-demographic, sex work, behavioural and sexual health characteristics of adolescent female sex workers, stratified by alcohol use and tobacco use in the past year (*N* = 310)

Characteristics	All women (***N*** = 310) n (%)	Abstain or occasional drinking (***N*** = 53; 17%) n (%)	Regular drinking (***N*** = 257; 83%) n (%)	Abstain or occasional smoking (***N*** = 174; 56%) n (%)	Regular smoking (***N*** = 136; 44%) n (%)
**Socio-demographic variables**					
Age, median years (IQR) ^a^	19(18–19)	19(18–19)	19(18–19)	19(18–19)	19(18–19)
Education level			**		
Illiterate or primary school only	89(29)	32(60)	57(22)	58(33)	31(23)
Middle school (Grade 7–9)	166(54)	16(30)	150(58)	89(51)	77(57)
High school or higher (Grade 10–12)	55(18)	5(10)	50(20)	27(16)	28(20)
Current marital status					
Never married or single	145(47)	22(42)	123(48)	89(51)	56(41)
Married or cohabiting	165(53)	31(59)	134(52)	85(49)	80(59)
Place of birth					
local	53(17)	9(17)	44(17)	29(17)	24(18)
Elsewhere	257(83)	44(83)	213(83)	145(83)	112(82)
**Sex work variables**					
Median weekly number of male clients (PM) (IQR) ^a^	2(1–3)	2(1–3)	2(1–3)	2(1–3)	2(2–3) *
Duration in sex work					
≤ half year	191(62)	34(64)	157(61)	117(67)	74(54)
> half year	119(38)	19(36)	100(39)	57 (33)	62(46)
Average monthly income from sex work	95(31)		**		
(past 3 months)^cd^	213(69)	30(57)	65(26)		**
≤360 EUR (low level)		23(43)	190(74)	66(38)	29(21)
> 360EUR (Middle to high level)				106(62)	107(79)
**Sexual behavioural variables**					
Age at sex debut, median years (IQR) ^a^	17(16–18)	16(15–18)	17(16–18)	16(16–18)	16(16–17) *
Coerced for forced sex at sex debut	70(23)	16(31)	54(21)	34(20)	36(27)
Median number of intimate male partner (PY) (IQR) ^a^	1(1–2)	1(1–2)	1(1–2)	1(1–2)	1(1–2)
Had any intimate male partner with illicit drug use	59(19)	1(2)	58(23)**	22(13)	37(27) **
Had sex without a condom while drunk (PM)	97(31)	7(13)	90(35)*	39(22)	58(43) **
Experienced sexual and gender-based violence (PY)	118(38)	6(11)	112(44)**	51(29)	67 (49) **
Inconsistent condom use with any male partners (PM)	224(72)	31(59)	193(75)*	118(68)	106(78) *
Unmet need for modern contraception	110(36)	9(17)	101(39) *	57(33)	53(39)
Utilization of health service for any STIs treatment^e^					**
Safe medical care	193(65)	35(71)	158(63)	120(73)	73(55)
Unsafe medical care	105(35)	14(29)	91(37)	44(27)	61(45)
Received HIV testing (PY)^f^	140(45)	33(64)	107(42)*	85(49)	55(40)
**Sexual health variables**					
Prior abortion	136(44)	12(23)	124(48)**	66(38)	70(52) *
Any self-reported symptom of STIs (PY)	205(66)	22(42)	183(71)**	93(53)	112(82) **
**Concurrent substance use**					
Illicit drug use ^b^	27(9)	1(2)	26(10)*	4(2)	23(17) **
Regular-alcohol drinker	257(83)	–	–	122(70)	135(99) **
Regular-tobacco smoker ^b^	136(44)	1(2)	129(50) **	**–**	**–**

*p*-value using Pearson’s Chi-squared tests, unless otherwise indicated: ^a^ Wilcoxon rank sum test, ^b^ Fisher’s exact test

*IQR* interquartile range, *PY* past year, *PM* past month

*p-value ≤0.05; ** *p*-value ≤0.001

^c^Average income was divided into three groups (low level, middle level and high level) according to local income level of general population in Kunming in 2012

^d^ Two responses were missing in the question of monthly income;^e^ Twelve responses were missing in the question of STIs treatment;^f^ One response was missing in the question of HIV testing

### Patterns of substance use

The overall prevalence of regular-tobacco smoking was 44% (136/310) among enrolled AFSWs. The illicit drug use was reported by 9% (27/310) of the participants. Not surprisingly, our study revealed a high prevalence of regular-alcohol drinking (83%, 257/310) among AFSWs (Table 1).

The prevalence of concurrent substance use is also identified in this study: a majority of illicit drug-using participants were also drinking alcohol (96%; 26/27) and smoking tobacco regularly (85%; 23/27); whilst a majority of AFSWs who regularly smoke tobacco were also regularly drinking alcohol (95%; 129/136) (Table 1). The 84% (259) of participants were concurrently using polysubstance (Table 2).

**Table 2:** Socio-demographic, sex work, behavioural and sexual health characteristics of adolescent female sex workers, stratified by illicit drug use and polysubstance use in the past year (*N* = 310)

Characteristics	Abstain (***N*** = 283; 91%) n (%)	illicit drug use (***N*** = 27; 9%) n (%)	Abstain (***N*** = 51; 16%) n (%)	Polysubstance use (***N*** = 259; 84%) n (%)
**Socio-demographic variables**				
Age, median years (IQR)^a^	19(18–19)	19(17–19)	19(18–19)	19(18–19)
Education level				**
Illiterate or primary school only	81(29)	8(30)	32(63)	57(22)
Middle school (Grade 7–9)	150(53)	16(59)	14(27)	152(59)
High school or higher (Grade 10–12)	52(18)	3(11)	5(10)	50(19)
Current marital status				
Never married or single	134(48)	11(41)	20(39)	125(48)
Married or cohabiting	149(52)	16(59)	31(61)	134(52)
Place of birth				
local	49(17)	4(15)	8(16)	45(17)
Elsewhere	234(83)	23(85)	43(84)	214(83)
**Sex work variables**				
Median weekly number of male clients (PM) (IQR)^a^	2(1–3)	2(2–4)	2(1–3)	2(1–3)
Duration in sex work				
≤ half year	176(62)	15(56)	32(63)	159(61)
> half year	107(38)	12(44)	19(37)	100(39)
Average monthly income from sex work^cd^		*		**
≤ 360 EUR (low level)	92(33)	3(11)	28(55)	67(26)
> 360EUR (Middle to high level)	189(67)	24(89)	23(45)	190(74)
**Sexual behavioural variables**				
Age at sex debut, median years (IQR)^a^	17(16–18)	16(16–17)	16(15–18)	17(16–18)
Coerced for forced sex at sex debut	55(19)	15(56)**	15(29)	55(21)
Median number of intimate male partner (PY) (IQR)^a^	1(1–2)	2(1–2)	1(1–2)	1(1–2)
Had any intimate male partner with illicit drug use	44(16)	15(56)**	1(2)	58(22)**
Had sex without a condom while drunk (PM)	86(30)	11(41)	7(14)	90(35)*
Experienced sexual and gender-based violence (PY)	99(35)	19(70)**	6(12)	112(43)**
Inconsistent condom use with any male partners (PM)	200(71)	24(89)*	30(59)	194(75)*
Unmet need for modern contraception	97(34)	13(48)	8(16)	102(39) **
Utilization of health service for any STIs treatment^e^		**		
Safe medical care	186(69)	7(27)	34(71)	159(64)
Unsafe medical care	86(31)	19(73)	14(29)	91(36)
Received HIV testing (PY)^f^	128(45)	12(44)	31(62)	109(42) *
**Sexual health variables**				
Prior abortion	12(23)	17(63)*	11(22)	125(48) **
Any self-reported symptom of STIs (PY)	22(42)	23(85)*	21(41)	184(71) **

*p*-value using Pearson’s Chi-squared tests, unless otherwise indicated: ^a^ Wilcoxon rank sum test, ^b^ Fisher’s exact test

*IQR* interquartile range, *PY* past year, *PM* past month

*p-value ≤0.05; ** *p*-value ≤0.001

^c^Average income was divided into three groups (low level, middle level and high level) according to local income level of general population in Kunming in 2012

^d^ Two responses were missing in the question of monthly income;^e^ Twelve responses were missing in the question of STIs treatment;^f^ One response was missing in the question of HIV testing

### Main characteristics of AFSWs who use substance

In comparison with abstainers or occasional alcohol-drinking AFSWs, regularly alcohol-drinking AFSWs were more likely to have middle school education (58% vs. 30%) and to gain higher income from sex work (74% vs. 43%), to be exposed to SGBV (44% vs. 11%) and illicit drug–using partners (23% vs. 2%), to engage in unprotected sex while drunk (35% vs. 13%) and inconsistent condom use with male partners (75% vs. 59%), to report unmet need for modern contraception (39% vs. 17%), prior abortion (48% vs. 23%) and symptom of STIs (71% vs. 42%), to use illicit drug (10% vs. 2%) and to regularly smoke tobacco (50% vs. 2%). Moreover they are less likely to access HIV testing service (42% vs. 64%) in the past year (Table 1).

In comparison with abstainers or occasionally tobacco-smoking AFSWs, participants who regularly smoke tobacco were less likely to enter sex work more than half-year duration (54% vs. 67%); they were more likely to have higher monthly income (79% vs. 62%), to experience sex debut at earlier adolescence (Interquartile Range: 16–17 vs. 17–18); to encounter illicit drug–using partners (17% vs. 2%) and SGBV (49% vs. 29%); to engage in unprotected sex while drunk (43% vs. 22%) and inconsistent condom use with male partners (78% vs. 68%); to report STIs symptoms (82% vs. 53%) and prior abortion (52% vs. 38%); to use unreliable health providers for STIs treatment (45% vs. 27%); to drink alcohol regularly (99% vs. 70%) and use illicit drug (17% vs. 2%) (Table 1).

In comparison with abstainers, illicit drug-using AFSWs were more likely to report higher monthly income (89% vs. 67%); to be exposed to forced sex debut (56% vs. 19%), illicit drug-using partners (56% vs. 16%) and SGBV (70% vs. 35%); to report inconsistent condom use with male partners (89% vs. 71%), prior abortion (63% vs. 23%) and symptom of STIs (85% vs. 42%); and to use unreliable health providers for STIs treatment (73% vs. 31%), (Table 2).

In comparison with abstainers, polysubstance-using AFSWs were more likely to have middle school education (59% vs. 27%) and higher monthly income (74% vs. 45%); to be exposed to illicit drug-using partners (22% vs. 2%) and SGBV (43% vs. 12%); to report inconsistent condom use with male partners (75% vs. 59%), unprotected sex while drunk (35% vs. 14%), unmet need for modern contraception (39% vs. 16%), prior abortion (48% vs. 22%) and STIs symptoms (71% vs. 41%). On the other hand, they are less likely to access HIV testing service (42% vs. 62%) in the past year (Table 2).

### Factors associated with regular-alcohol drinking

Table 3 summarized the results of final multivariable logistic regression model of factors associated with regular-alcohol drinking after adjusting for all factors associated in bivariate analysis. AFSWs who had middle and high school education (7-9th grade: adjusted odds ration ‘AOR’ = 5.33; 95%CI = 2.37–12.00, 10-12th grade: AOR = 3.27; 95%CI = 1.01–10.59), had higher monthly income (AOR = 3.26; 95%CI = 1.50–7.10), experienced of SGBV (AOR = 4.29; 95%CI = 1.55–11.85) and prior abortion (AOR = 2.70; 95%CI = 1.17–6.22), and regular-tobacco smoking (AOR = 42.11; 95%CI = 5.54–320.36) were associated with significantly increased odds of regular-alcohol drinking.

**Table 3:** Multivariable logistic regression analysis of factors associated with regular-alcohol drinking behaviour in the past year among adolescent female sex workers (*N* = 310)

Factors	Regular-alcohol drinking				
	n/N (%)	Crude OR (95% CI)	***p***-value	Adjusted OR^**a**^ (95% CI)	***p***-value
Education level					
Illiterate or primary school only	57/89(83)	1.0		1.0	< 0.001
Middle school (Grade 7–9)	150/166(90)	5.26(2.58–10.73)		5.33(2.37–12.00)	0.049
High school or higher (Grade 10–12)	50/55(91)	5.61(1.93–16.34)	< 0.001	3.27(1.01–10.59)	
Average monthly income from sex work					
≤ 360 EUR (low level)	65/95 (68)	1.0		1.0	
> 360 EUR (Middle level to high level)	190/213 (89)	3.81(2.02–7.19)	< 0.001	3.26(1.50–7.10)	0.003
Had any intimate male partner with illicit drug use					
No	199/251(79)	1.0			
Yes	58/59(98)	15.15(1.96–117.03)	< 0.001		
Had sex without a condom while drunk (PM)					
No	167/213(78)	1.0			
Yes	90/97(93)	3.54(1.51–8.29)	0.002		
Experienced sexual and gender-based violence (PY)					
No	145/192(76)	1.0		1.0	
Yes	112/118(95)	6.05(2.42–15.11)	< 0.001	4.29(1.55–11.85)	0.005
Inconsistent condom use with any male partners (PM)					
No	64/86(74)	1.0			
Yes	193/224(86)	2.14(1.15–3.99)	0.014		
Unmet need for modern contraception					
No	156/200(78)	1.0			
Yes	101/110(92)	3.17(1.46–6.86)	0.002		
Received HIV testing (PY)					
Yes	107/140(76)	1.0			
No	150/169(89)	2.43(1.30–4.55)	0.004		
Prior abortion					
No	133/174 (76)	1.0		1.0	
Yes	124/136(91)	3.19(1.58–6.43)	< 0.001	2.70(1.17–6.22)	0.020
Any self-reported symptom of STIs (PY)					
No	74/105(71)	1.0			
Yes	183/205(89)	3.49(1.86–6.53)	< 0.001		
Illicit drug use					
No	231/283(82)	1.0			
Yes	26/27(96)	5.85(0.76–44.8)	0.053		
Tobacco use					
Abstainer occasional smoking	122/174(70)	1.0		1.0	
Regular smoking	135/136(99)	57.54(6.61–500.48)	< 0.001	42.11(5.54–320.36)	< 0.001

In the final logistic regression model: Pseudo R2 = 0.3866; *P* < 0.001; Akaike information criterion =187.49

*CI* confidence interval, *OR* odds ratio in the bivariate logistic regression model

^a^Adjusted odds ratio in the final logistic regression model

### Factors associated with regular-tobacco smoking

Four factors were detected to associate with AFSWs’ regular-tobacco smoking in the final multivariate logistic regression models (Table 4). AFSWs had the unprotected sex while drunk had a 1.8-fold odds of regular-tobacco smoking compared to those who did not (95%CI = 1.04–3.15); AFSWs with STIs symptoms had a 2.8-fold odds of regular-tobacco smoking compared to the AFSWs who did not (95%CI = 1.55–5.01); AFSWs who used illicit drug was associated with a greater than 6 times of the odds to regular smoking (95%CI = 2.00–23.36); similarly, AFSWs who regularly drink alcohol had a 40-fold higher odds of regular-tobacco smoking (95%CI = 5.43–299.76).

**Table 4:** Multivariable logistic regression analysis of factors associated with regular-tobacco smoking behaviour in the past year among adolescent female sex workers (*N* = 310)

Factors	Regular-tobacco smoking				
	n/N (%)	Crude OR (95% CI)	***p***-value	Adjusted OR^**a**^ (95% CI)	***p***-value
Duration in sex work					
≤ half year	74/191(39)	1.0			
> half year	62/119(52)	1.71(1.07–2.75)	0.021		
Average monthly income from sex work					
≤ 360 EUR (low level)	29/95 (31)	1.0			
> 360 EUR (Middle level to high level)	107/213(50)	3.81(2.02–7.19)	< 0.001		
Age at sex debut					
18–19 years	25/82(31)	1.0			
12–15 years	87/175(50)	2.25(1.28–3.97)	0.040		
16–17 years	24/53(46)				
Had any intimate male partner with illicit drug use					
No	99/251(39)	1.0			
Yes	37/59(63)	2.58(1.42–4.69)	0.001		
Had sex without a condom while drunk (PM)					
No	78/213(37)	1.0		1.0	
Yes	58/97(60)	2.57(1.55–4.27)	< 0.001	1.81(1.04–3.15)	0.035
Experienced sexual and gender-based violence (PY)					
No	69/192(36)	1.0			
Yes	67/118(57)	6.05(2.42–15.11)	< 0.001		
Inconsistent condom use with any male partners (PM)					
No	30/86(35)	1.0			
Yes	106/224(47)	1.68(1.00–2.82)	0.049		
Utilization of health service for any STIs treatment^!!^					
Safe medical care	73/193(38)	1.0			
Unsafe medical care	61/105(58)	2.28(1.39–3.74)	< 0.001		
Prior abortion					
No	66/174 (38)	1.0			
Yes	70/136(51)	1.73(1.09–2.75)	0.017		
Any self-reported symptom of STIs (PY)					
No	24/105(23)	1.0		1.0	
Yes	112/205(55)	4.06(2.32–7.11)	< 0.001	2.79(1.55–5.01)	0.001
Illicit drug use					
No	113/283(40)	1.0		1.0	
Yes	23/27(85)	8.65(2.80–26.72)	< 0.001	6.84(2.00–23.36)	0.002
Alcohol use					
Abstainer occasional drinking	1/53(2)	1.0		1.0	
Regular drinking	135/257(53)	57.54(6.62–500.48)	< 0.001	40.35(5.43–299.76)	< 0.001

In the final logistic regression model: Pseudo R2 = 0.23; *P* < 0.001; Akaike information criterion =339.33

*CI* confidence interval, *OR* odds ratio in the bivariate logistic regression model

^a^Adjusted odds ratio in the final logistic regression model

!!Twelve responses were missing in the question of STI treatment

### Factors associated with illicit drug use

In multivariate analysis, AFSWs who had an illicit drug using- intimate partner (AOR =5.77; 95%CI = 2.33–14.29), experienced forced sexual initiation (AOR = 4.41; 95%CI = 1.77–11.06), utilization of unsafe medical providers for STIs treatment or no treatment at all (AOR = 5.98; 95%CI = 2.26–15.85) were associate with increased odds of illicit drug use (Table 5).

**Table 5:** Multivariable logistic regression analysis of factors associated with any illicit drug use in the past year among adolescent female sex workers (*N* = 310)

Factors	Illicit drug use				
	n/N (%)	Crude OR (95% CI)	***p***-value	Adjusted OR^**a**^ (95% CI)	***p***-value
Average monthly income from sex work^!^					
≤ 360 EUR (low level)	3/95(3)	1.0			
> 360EUR (Middle to high level)	24/213(11)	3.89(1.12–13.44)	0.020		
Coerced for forced sex at sex debut					
No	12/240(5)	1.0		1.0	
Yes	15/70(21)	5.18(2.23–12.01)	< 0.001	4.41(1.77–11.06)	< 0.001
Had any intimate male partner with illicit drug use					
No	12/251(5)	1.0		1.0	
Yes	15/59(25)	6.79(2.87–16.08)	< 0.001	5.77(2.33–14.29)	0.002
Experienced sexual and gender-based violence (PY)					
No	8/192(4)	1.0			
Yes	19/118(16)	4.41(1.82–10.66)	< 0.001		
Inconsistent condom use with any male partners (PM)					
No	3/86(3)	1.0			
Yes	24/224(11)	3.32(0.96–11.44)	< 0.001		
Utilization of health service for STI treatment					
Safe medical care	7/193(4)	1.0		1.0	
Unsafe medical care	19/105(18)	5.87(2.31–14.93)	< 0.001	5.98(2.26–15.85)	< 0.001
Prior abortion					
No	10/174(6)	1.0			
Yes	17/136(1)	2.34(1.03–5.34)	0.037		
Any self-reported symptom of STIs (PY)					
No	4/105(4)	1.0			
Yes	23/205(11)	3.19(1.06–9.58)	0.029		

In the final logistic regression model: Pseudo R2 = 0.25; *P* < 0.001; Akaike information criterion =140.08

*CI* confidence interval, *OR* odds ratio in the bivariate logistic regression model

^a^Adjusted odds ratio in the final logistic regression model

!Two responses were missing in the question of monthly income

### Factors associated with polysubstance use

AFSWs who had middle and high school education (7-9th grade: AOR = 6.81; 95%CI = 3.09–15.01, 10-12th grade: AOR = 4.26; 95%CI = 1.39–13.00), had higher monthly income (AOR = 2.94; 95%CI = 1.40–6.14), experienced of SGBV (AOR = 5.74; 95%CI = 2.19–15.05), had unmet need for modern contraception (AOR = 3.47; 95%CI = 1.44–8.40), reported prior abortion (AOR = 2.95; 95%CI = 1.31–6.64) were associated with a significantly increased odds of polysubstance use (Table 6).

**Table 6:** Multivariable logistic regression analysis of factors associated with polysubstance use in the past year among adolescent female sex workers (*N* = 310)

Factors	Polysubstance use				
	n/N (%)	Crude OR (95% CI)	***p***-value	Adjusted OR^**a**^ (95% CI)	***p***-value
Education level					
Illiterate or primary school only	57/89(64)	1.0		1.0	
Middle school (Grade 7–9)	152/166(92)	6.10(2.90–12.83)		6.81(3.09–15.01)	< 0.001
High school or higher (Grade 10–12)	50/55(91)	5.61(1.93–16.34)	< 0.001	4.26(1.39–13.00)	0.011
Average monthly income from sex work					
≤ 360 EUR (low level)	67/95(71)	1.0		1.0	
> 360EUR (Middle to high level)	190/213(89)	3.45(1.83–6.52)	< 0.001	2.94(1.40–6.14)	0.004
Had any intimate male partner with illicit drug use					
No	201/251(80)	1.0			
Yes	58/59(98)	14.43(1.87–111.24)	< 0.001		
Had sex without a condom while drunk (PM)					
No	169/213(79)	1.0			
Yes	90/97(93)	3.35(1.43–7.84)	0.003		
Experienced sexual and gender-based violence (PY)					
No	147,192(77)	1.0		1.0	
Yes	112/118(95)	5.71(2.29–14.27)	< 0.001	5.74(2.19–15.05)	< 0.001
Inconsistent condom use with any male partners (PM)					
No	65/86(76)	1.0			
Yes	194/224(87)	2.09(1.11–3.93)	0.019		
Unmet need for modern contraception					
No	157/200(79)	1.0		1.0	
Yes	102/110(93)	3.49(1.55–7.85)	0.001	3.47(1.44–8.40)	0.006
Received HIV testing (PY)					
Yes	109/140(78)	1.0			
No	150/169(89)	2.25(1.20–4.22)	0.009		
Prior abortion					
No	134/174(77)	1.0		1.0	
Yes	125/136(92)	3.39(1.64–7.01)	< 0.001	2.95(1.31–6.64)	0.009
Any self-reported symptom of STIs (PY)					
No	75/105(72)	1.0			
Yes	184/205(90)	3.50(1.85–6.63)	< 0.001		

In the final logistic regression model: Pseudo R2 = 0.29; *P* < 0.001; Akaike information criterion =210.80

*CI* confidence interval, *OR* odds ratio in the bivariate logistic regression model

^a^Adjusted odds ratio in the final logistic regression model

### The needs for comprehensive sexual and reproductive health services

In this study, we identified comprehensive SRH service needs among AFSWs. The need of free and available HIV/STIs pre-and post-test counselling services was reported by the majority of respondents (69%; 214/309), which was followed by an access to free condom and education of proper condom use (61%; 189/309) (Table 5). Other significant needs including affordable and safe abortion, maternity and gynaecology services (45%; 139/309), STIs diagnosis and treatment services (42%; 129/309), easy access to counselling (42%; 129/309) and available contraception methods services (21%; 65/309) (data not shown in table).

Our findings were brought to light that 65% (193/298) of adolescent women sought STIs treatment from a safe healthcare providers; while other 35% (105/298) were from untrained providers or having a self-medication, or no treatment at all, of whom 87% (91/105) were using polysubstance (Tables 2).

## Discussion

To our knowledge, this is first study to report the high prevalence of concurrent polysubstance use including alcohol, illicit drug and cigarettes, as well as their relations to multiple SRH risks among AFSWs in China. Furthermore, the basic data of the AFSWs’ holistic SRH needs are essential for designing and improving the intervention programme.

### Developing specific intervention to mitigate the alcohol and tobacco-related harms

Several studies confirmed the harm arising from alcohol use as an internationally public health challenge for adolescents. For example, alcohol use disorders explained 5% of all mental, neurological, and substance use disorder burden in China [26]. Available data also showed that, heavier alcohol consumption in late adolescence continues into adulthood and is also associated with alcohol problems including alcohol use disorder [27]. The Healthy China Action Plan (2019–2030) [28] is a new and ambitious initiative to achieve the better health for everyone. This blueprint sets targets for tobacco control, including a reduction in the rate of smoking to 24.5% by 2022 and 20% by 2030; however, there is no specific target towards alcohol consumption control.

Moreover, traditional HIV or SRH intervention targeting FSWs rarely addresses the harm of alcohol, let alone focusing on AFSWs. At a practical level for intervention, understanding AFSWs’ work environment and economic condition has the implications to intervention design. Binge drinking is common in many sex work venues, where alcohol or varieties of alcoholic beverages outlets financially benefit sex workers, alcohol consumption of FSWs and their clients become a significant source of income [12]. Our study clearly demonstrated the high prevalence (83%) of heavier alcohol intake among AFSWs, the rate was much higher than general adolescent population (7.3%) in China [29]. Most AFSWs were new rural-to-urban migrants which face to economic pressures, the higher frequency of situational and environmental alcohol assumption meant the higher income obtained; meanwhile it implies a deeply intertwined link between sex work and alcohol consumption for AFSWs, in turn put them at risk of alcohol use disorder, unwanted or unsafe sex, violence and HIV/STIs.

Tobacco use is another major public health issue among AFSW. Individuals struggling financially face greater incentivizes to smoke as a coping response than those who are financially secure [30], in this study, given the striking rates of regular smoking among AFSWs, which is much higher than their school-based adolescent counterparts (44% vs. 14.7%) in national data [31], there is a need for integrating counselling about the risks of alcohol and tobacco prevention for AFSWs, especially pay attention to new rural-to-urban female migrants in young age; other interventions focused on financial security and welfare, job skills training, housing and legal services should be available in public health campaigns targeting marginalized youth and adolescents.

From a perspective of the population-based approach, in China, the effectiveness of current tobacco control policy in general adolescents and youth needs to be strengthened. China has been a signatory country to the WHO Framework Convention on Tobacco Control since 2006, but China is the world’s largest producer with a high-burden of tobacco-use and, one of five focus countries for the Bloomberg Initiative to Reduce Tobacco Use. Cigarettes are cheap in China, and have become more affordable over time; meanwhile alcohol and cigarettes are easy accessed for adolescents, these are important factors influencing the adolescents’ smoking and drink prevalence [31]. Current health policy and plan should advance controlling overall alcohol and tobacco consumption to mitigate the harms from adolescents and young adults, the pricing, restriction of advertisement and purchasing age are most cost-effective strategies, which lie at the legislation, taxation and strict implementation of policies.

The absence of universal comprehensive sexuality and substance prevention education is another important gap needed to be addressed in China [32]. Despite higher average levels of education were found to associate with benefits of adolescent health and wellbeing in countries of low and middle income [4], whereas our analysis demonstrated that more schooling attained did not protect them from heavier alcohol consumption and polysubstance use in comparison to participants with lower education level (illiterate or primary education), the result was contrary to our hypothesis. This should not be taken as evidence that education is not the protective factor for adolescents’ health and wellbeing, but the finding may reflect the fact that the inadequate or inappropriate education regarding substance prevention and sexuality failed to empower female adolescents with necessary knowledge and skills before them dropping out of school and entering into the workplaces. These findings converge to suggest the importance of integrating substance prevention and SRH promoting into primary and secondary school settings. This approach should be tailored as local context-related, age-related and gender-sensitive, particularly in areas with high prevalence of and health burden related to illicit drug, alcohol and tobacco use, such as Yunnan Province.

### Supporting links between harm reduction and SRH care services

The findings of this study confirm that AFSWs with illicit drug use were 5.9 times more likely to use unreliable health providers for STIs treatment than those without illicit drug use; moreover, of the 105 participants sought medical treatment from unsafe providers, or used self-medication, or no treatment at all for their STIs symptoms, the majority (87%; 91/105) of them using polysubstance. For illicit drug use, Chinese current registration system of the public policy sector meant that adolescent incarcerators were all registered as drug users and under the surveillance of police for as long as 3 years after their last positive drug test. Therefore, they could be questioned or tested for drugs by police at any time in this surveillance period [33]. In addition, the criminal records (arrested as a drug user) will brand them for life (drive license and identity card). The criminalization, policies and practices are obvious barriers of applying for a job and social welfare, and can also foster and perpetuate adolescent’ vulnerability to social exclusive and discrimination in healthcare settings. Social taboos against sexually active adolescents, fear of disclosure of illegal behaviours, fear of prejudice from healthcare workers, inconvenient time schedule and locations of health facilities, lack of medical insurance and adequate information, are all likely to deter or discourage AFSWs from seeking the SRH and substance treatment from qualified healthcare service providers [34].

In this study, the triple jeopardy facing AFSW, these include concurrent substance use, combined SRH risks and less access to qualified healthcare services. This reality highlights the importance of interweaving key programmatic elements of harm reduction with SRH service including HIV/STIs intervention, for example: early warning systems for substance abuse, communication strategies of prevention, guidelines for managing alcohol and other substance abuse -related disorders, as well as training for health workers to eliminate discrimination towards AFSWs.

### Risky intimate partners contribute to AFSWs’ substance use and SRH risks

There has been clear and consistent evidence of an association between heavy drink or drinking large amounts per occasion and intimate partner violence [35]. A review found that alcohol use by FSWs and their clients reduced safer sex and social interactions, but this area remains under researched [36]. A line of research showed traumatic events, such as experiencing violent victimization, has a significant impact on adolescents’ poor mental health and female first cigarette use behaviour [30]. Our findings from bivariate logistic regression analysis examined the significant association between risky partners and AFSWs’ substance use, reflecting the gendered issue. Our previous study also showed illicit drug using-AFSWs were more likely to select drug-using intimate partner, potential reasons could be drug supply and/or economic gains; or may be invited or hired to share partners’ drugs [34, 37]; in this situation, AFSWs may lose control of dose, use pattern and use venue, consequently inhibiting their ability to negotiate safe sex, which in turn expose them to higher SRH risks, particularly HIV/STIs [34]. Thus, effective substance use interventions need to address low-control or unequal power relationships between risky intimate partners and AFSWs. Peer support and youth’s rights-based efforts can join forces to draw attention to their unique circumstances and protect them from risky intimate partners.

### Limitation and priorities for further research

These findings need to be interpreted in light of certain limitations. Firstly, the cross-sectional nature of this study prevents the attribution of causality, thus we could not assess the temporal sequence. Secondly, given the severe penalties for illicit drug use, fear of disclosure of illicit drug use may have resulted in the cases of illicit drug use and drug-using partners being underreported. In consolidating future directions, there is valuable for research evaluating the effectiveness and efficiency of evidence-based interventions from integrated healthcare settings that meet vulnerable adolescent women’ needs.

## Conclusions

SRH and rights are, in turn, fundamental for adolescent women’s wellbeing and full participation in society [38], and are integral to the core targets of *Global Strategy for Women’s, Children’s, and Adolescents’ Health* (2016–2030) [3] and the Healthy China Action Plan (2019–2030) [28]. As a highly marginalized and vulnerable population, AFSWs have an equal chance to survive, thrive and contribute to the transformative change envisioned by social development goals. Achieving the goals will require legal and policy reforms to address structural barriers in a systematic way. A lack of integration of intervention delivery platforms has hindered service utilization and efficacy to deal with AFSWs’ overlapping vulnerabilities. This study emphasises that coordinated efforts are needed to integrate SRH promotion and harm reduction service action across sectors, and not only fragmented measures, our suggestions are not merely based on assumptions, but informed by evidence.

## Data Availability

A minimal dataset is available upon request.

## Abbreviations

AFSWs: Adolescent female sex workers
AIC: Akaike information criterion
AOR: Adjusted odds ratio
DALYs: Disability-adjusted life-years
NCDs: Non-communicable diseases
SGBV: Sexual and gender-based violence
SRH: Sexual and reproductive health
STIs: Sexually transmitted infections
